# Overview of Oxidative Stress in Systemic Lupus Erythematosus

**DOI:** 10.3390/antiox14030303

**Published:** 2025-03-01

**Authors:** Ancuta Lupu, Gabriela Stoleriu, Alin Horatiu Nedelcu, Sara Nadeea Perju, Cristina Gavrilovici, Ginel Baciu, Cristina Maria Mihai, Tatiana Chisnoiu, Ionela Daniela Morariu, Ecaterina Grigore, Shwan Karwan Shawais, Delia Lidia Salaru, Ninel Revenco, Vasile Valeriu Lupu

**Affiliations:** 1 Pediatrics, “Grigore T. Popa” University of Medicine and Pharmacy, 700115 Iasi, Romania; ancuta.ignat1@umfiasi.ro (A.L.); cri.gavrilovici@umfiasi.ro (C.G.); vasile.lupu@umfiasi.ro (V.V.L.); 2 Clinical Medical Department, Faculty of Medicine and Pharmacy, “Dunarea de Jos” University of Galati, 800008 Galati, Romania; gabriela.stoleriu@ugal.ro (G.S.); ginel.baciu@ugal.ro (G.B.); 3 Faculty of Medicine, “Grigore T. Popa” University of Medicine and Pharmacy, 700115 Iasi, Romaniashwansaddik@gmail.com (S.K.S.); delia.salaru@umfiasi.ro (D.L.S.); 4 Pediatrics, Faculty of Medicine, “Ovidius” University, 900470 Constanta, Romania; cristina_mihai@365.univ-ovidius.ro (C.M.M.); tatiana.chisnoiu@365.univ-ovidius.ro (T.C.); 5 Faculty of Pharmacy, “Grigore T. Popa” University of Medicine and Pharmacy, 700115 Iasi, Romania; ionela.morariu@umfiasi.ro; 6 Pediatrics, “Nicolae Testemitanu” State University of Medicine and Pharmacy, 2004 Chisinau, Moldova; ninel.revenco@usmf.md

**Keywords:** systemic lupus erythematosus, reactive oxygen species, NETosis, mTOR pathway activation, T-cell differentiation

## Abstract

Systemic lupus erythematosus (SLE) is an autoimmune disease that is frequently diagnosed in female patients, caused by multiple interacting factors. It has a complex pathogenesis which can affect almost any organ, from the kidneys to the cardiovascular, pulmonary, neurological, osteoarticular, and hematological systems. The present narrative review seeks to elucidate the role of reactive oxygen species (ROS) in the pathogenesis of SLE. The central question guiding this study is to what extent these serum protein modifications correlate with disease activity and organ damage in SLE. It is characterized by the decreased apoptosis and increased necrosis of T cells and the NETosis of granulocytes. Given the impact of an SLE diagnosis on one’s life, this narrative review aims to evaluate the intricacies of oxidative stress and its relevance to the pathogenesis and treatment of the disease. Topics such as understanding processes of oxidative stress, their damaging pathways, oxidative stress biomarkers, and their role in the future assistance of clinical decisions will be discussed in the article. The accurate determination of biomarkers is taught to improve both the diagnosis and the management of the disease, while antioxidant therapy may open a new door for the treatment.

## 1. Introduction

The multifactorial autoimmune disease systemic lupus erythematosus (SLE) has been shown to have a complex pathogenesis, with increased autoantibody production being the hallmark of the condition. Oxidative stress is defined as the imbalance between the synthesis and neutralization of reactive oxygen species (ROS) [1].

Autoimmune diseases are known to result from a combination of genetic and environmental factors [2,3]. The most important cells in the evolution of autoimmune diseases appear to be lymphocytes, especially T and B cells, but dendritic cells, macrophages, and neutrophils also have an important role in SLE [1,4]. Naturally, lymphocytes are activated by foreign antigens, not by a person’s own nucleic acids, a trait known as immunological tolerance. Deoxyribonucleases (DNases) are the enzymes which normally break down human DNA, and the absence of DNase1 is an essential biomarker for the beginning of autoimmune diseases [5,6]. There are two ways in which the immune system eliminates self-reactive lymphocytes: central tolerance, destroying naive T cells in their initial development in the bone marrow and thymus; and peripheral tolerance, which eliminates self-reactive lymphocytes in peripheral tissues. Neutrophils are pivotal effectors in the immune response to infection, responsible for the synthesis and release of ROS and the release of microbicidal molecules [6,7,8]. Dendritic cells are antigen-presenting cells that pickup antigens from the body and present them to naive T cells, partially stimulating their activation [9].

T-cell defects result in aberrant immune responses which partially explain the inflammatory pathology and comorbidities of SLE. Some of the main consequences of the disturbed immune response include persistent inflammation, chaotic antibody production, and the release of cytokines. The increased production of cytokines such as IL-17 and IL-21 in the blood has been demonstrated to lead to increased T-cell production. The increased presence of T cells, in turn, leads to an elevated probability of B cells presenting intranuclear complexes to T cells, via MHCII class molecules. Toll-like receptors interact with DNA and RNA, leading to the activation of these B cells, with the resultant mature B cells then acquiring the ability to produce autoantibodies which are pathogenic and cause organ damage through immune complex (IC) deposition, together with complement and neutrophil activation. The binding of antibodies to their cognate antigen results in the formation of ICs, which serve as potent stimulants for immune cells. Furthermore, the levels of autoantibodies exhibit a direct correlation with the severity of the disease and are considered the most significant diagnostic markers for SLE (Table 1) [8,9,10,11,12].

In SLE, impaired B cell clearance and improper apoptotic body disposal contributes to the alteration of the autoantigens’ clearance. This promotes IC formation and, as a result, classifies SLE as a prototype of type III hypersensitivity [16,17,18].

In type III hypersensitivity, ICs are formed and they deposit on blood vessel walls. Because the ICs are small, they are less immunogenic, so macrophages are less attracted to them. As a result, they reside and travel through the bloodstream for a longer period, which explains why in SLE there are deposits of ICs attached to the basement membrane. Once deposited, the ICs activate the complement system, a family of nine proteins (C1–C9). Three compounds of this family—C5a, C4a, and C3a—are anaphylatoxins, which increase vascular permeability and the level of chemokines, meaning they recruit other cells, like neutrophils, to the site of inflammation. Once neutrophils are attracted, they try to phagocytize the ICs but rarely can, and so they degranulate and cause vasculitis. Blood vessel inflammation and tissue necrosis results due to lysosomal enzymes and ROS that reside in their granules. Thus, more cells are destroyed and more DNA is released, repeating the vicious cycle [16,17,18].

In order to diagnose SLE, there are a series of clinical and immunological criteria which must be met. ACR1997 includes 11 criteria from which, if 4 or more are met, a diagnostic can be drawn (Table 2).

SLE manifests more severely in children than in adults. The condition is characterized by a higher incidence of malar rashes, nephritis, pericarditis, hematologic abnormalities, hepatosplenomegaly, and a faster pace of complication development [21].

Nowadays, the main criteria for diagnosing SLE are based on the SLICC (Systemic Lupus International Collaborating Clinics Criteria), asSLICC-2012 could achieve an earlier diagnosis. For 1997 ACR, there needs to be four or more criteria for a positive diagnosis, whereas for 2012 SLICC, one clinical and one paraclinical criteria or confirmed lupus nephritis (LN) with positive ANA or anti-DNA antibodies can already diagnose SLE.

According to Table 3, SLICC-2012 showed a better early diagnostic ability. Furthermore, SLICC-2012 had an excellent diagnostic accuracy with values of 95.0%, compared with ACR-1997 at 89.6%. The diagnostic rates of ACR-1997 and SLICC-2012 were 82.1%, and 98.0%, respectively. This study further investigated the accuracy of the ACR-1997 and SLICC-2012 classification criteria. Of the total 352 patients with SLE, 289 (82.1%) and 345 (98.3%) fulfilled the criteria for ACR-1997 and SLICC-2012, respectively [19].

Given the impact of an SLE diagnosis on one’s life, this narrative review aims to evaluate the intricacies of oxidative stress and its relevance to the pathogenesis and treatment of the disease. Topics such as understanding the processes of oxidative stress, their damaging pathways, oxidative stress biomarkers, and their role in the future assistance of clinical decisions will be discussed in the article. The accurate determination of biomarkers is taught to improve both the diagnosis and the management of the disease, while antioxidant therapy may open a new door for its treatment. To this, we add a summary presentation of the general data and therapeutic lines regarding the management of SLE. Moreover, we focus on the complications caused by oxidative stress compounds such as atherosclerosis, nephritis, and other autoimmune diseases. To ensure the up-to-date relevance of the information, a thorough literature review was conducted on recent articles published in international databases including PubMed, Scholar, and Web of Science. We bring forward both the classic theories and treatments and the most recent clinical studies which focus not only on the treatment of inflammation but on neutralizing oxidative stress compounds.

## 2. Understanding Oxidative Stress

In broad terms, molecular mimicry elicits an immune response, thereby impeding the capacity to differentiate between the host and the pathogen, this leads to the formation of pathogenic ICs [18]. These complexes, in turn, have been shown to mediate tissue damage in SLE. T lymphocytes that recognize most self-antigens expressed throughout the body are inactivated or eliminated centrally during development in the thymus. Infections tend to cause inflammation and the release of inflammatory cytokine molecules, like interferon-gamma, and enter the immune system through the lymphatic system to reach the lymph nodes. Therefore, the lymph node is specialized for presenting foreign antigens to generate immune responses. A body of research has emerged in recent years to support a causal link between elevated oxidative stress and disease activity, as well as tissue damage in autoimmune diseases. Oxidative stress contributes to the onset of inflammatory and cellular defects in the pathogenesis of SLE. The presence of inflammatory and cellular markers, including oxidative modifications of DNA, result in modifications in the structural compounds of the body such as lipids and proteins. This contributes to immune system dysregulation and leads to an aggressive autoimmune attack through molecular mechanisms such as mTOR pathway activation, elevated NETosis, and increased T-cell differentiation. The contemporary treatment of SLE relies primarily on the use of immunosuppressants. However, pharmacological approaches targeting oxidative stress have emerged as a promising avenue for treating patients with SLE [32].

**Figure 1:**
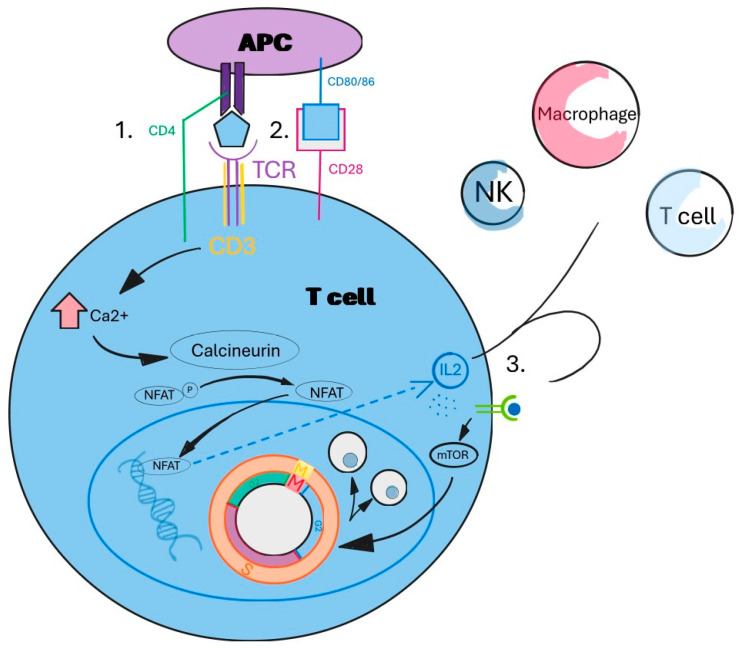
Activation and differentiation of CD4+ T cell.

It has been described that neutrophils undergo a process of cell death called NETosis, which is distinct from necrosis and apoptosis. NETosis is characterized by the sequential dissolution of inner membranes, followed by chromatin decondensation, and the subsequent release of these components into the extracellular space [23]. These webs of chromatin fibers, termed neutrophil extracellular traps (NETs), encompass histones, antimicrobial peptides, and oxidant-generating enzymes, including myeloperoxidase (MPO), neutrophil elastase, NOX, and NOS. This process is primarily initiated by the accumulation of ICs within the blood vessel. As demonstrated in the relevant literature, these structures have been identified as playing a pivotal role in a multitude of pathological states. This is due to the fact that they have the capacity to induce tissue damage, atherosclerosis, and thrombosis. NETosis can occur due to the release of ROS, with a vicious cycle being produced. ROS leads to morphological changes and can induce cell membrane dissolution. The close relationship between oxidative stress and NETosis suggests that therapies targeting oxidative stress may also reduce NETosis, leading to enhanced clinical outcomes for patients with chronic and autoimmune pathologies [33,34]. mTOR, a serine/threonine kinase and a central regulator of T-cell activation, differentiation, and homeostasis, is a key factor in the development of NETosis [35]. Researchers have identified excessively high concentrations of NO to be crucial upstream checkpoints of mTOR activation in T cells, which efficiently promote effector CD4+ T-cell activation and subsequent differentiation (Figure 1).

mTOR has been localized to endosomes along with the traffic regulator Rab4A which is a GTPase. Rab4A is overexpressed in the T cells of patients with SLE. This overexpression precedes mTOR activation, ANA production, and disease onset in SLE. Rab4A restrains mitophagy and promotes the accumulation of oxidative-stress-generating mitochondria [36]. mTOR constitutes the catalytic subunit of two distinct protein complexes: mTOR complex 1 (mTORC1) and 2 (mTORC2). Previous studies have indicated that mTORC1 activity is increased in individuals with SLE, thereby enhancing the production of the cytokine interleukin IL2. This, in turn, has been demonstrated to stimulate the proliferation of other lymphocytes and to reduce the number of regulatory T cells (Treg) in the body. The prevailing data largely corroborate the hypothesis that both mTORC1 and mTORC2 suppress the differentiation of effector T cells in FOXP3+ Treg cells, and that mTORC1 facilitates the expansion of pro-inflammatory lymphocyte T helper cells, while mTORC2 drives B-cell activation and autoantibody synthesis. It has been demonstrated that circulating Treg cells decrease during SLE flares, and that their immune suppressive function in lupus patients is weakened. It is suggested that therapeutic interventions with rapamycin (sirolimus) may prevent the depletion of these cells [35,37].

**Figure 2:**
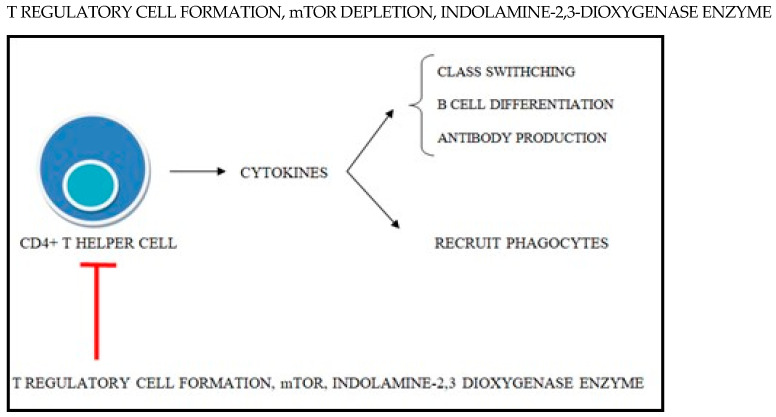
Peripheral tolerance mechanisms that aim at shutting down CD4+ T cells.

T helper cells produce cytokines which have major roles in B-cell differentiation, antibody production, and recruiting phagocytes at the inflammation site. It is evident that a significant proportion of the tolerance mechanisms are designed to induce the shutdown of CD4+ T cells (Figure 2) [35].

Regulatory T cells can inhibit the responses of all other immune cells expressing high levels of cytotoxic T lymphocyte-associated antigen 4 (CTLA4) and can also produce anti-inflammatory cytokines such as TGF beta and IL-10. Regulatory T-cell populations were depleted in patients with SLE compared with healthy controls. The mature T cell, after being activated by the antigen, can rarely respond strongly to self-antigens, in which case, there is an increase in the transcription of FOXP3, a factor which guides their development into regulatory T cells. Fully developed T regulatory cells express CTLA4 on their surface which, when signaled, induces the secretion of the enzyme IDO in dendritic cells. This IDO breaks down tryptophan and results in a local “immune privileged” microenvironment deprived of this essential amino acid. This is sensed by T lymphocytes which can stop CD8 T cells from proliferating and acting as cytotoxic T cells, so that the nearby tissue is protected from an attack by the immune system. CTLA4 binds to and inhibits B7 molecules on the APC and, as a result, it limits their co-stimulation properties, leaving T cells incompletely activated. Also, T regulatory cells express high levels of IL2 and adenosine receptors on their surface, depleting the availability of IL2 and adenosine compounds essential for the proliferation of other T lymphocytes [37,38,39,40,41,42,43,44,45,46,47,48].

## 3. Biomarkers of Oxidative Stress in Systemic Lupus Erythematosus

The sources of ROS can be categorized as either exogenous or endogenous. The term ‘exogenous’ is used in this context to denote sources of ROS that are derived from external factors, including ultraviolet radiation, exposure to chemicals, and viral or bacterial infections. Conversely, the term “endogenous” refers to sources of ROS formation that occur within the cell, such as in mitochondrial and organelle structures, including the endoplasmic reticulum [49,50].

Molecular oxygen is integral to the process of oxidative phosphorylation, in which mitochondria oxidize nutrients and utilize the energy released in the process to synthesize adenosine triphosphate (ATP). However, this vital function is connected to the production of ROS which are highly toxic [50].

**Figure 3:**
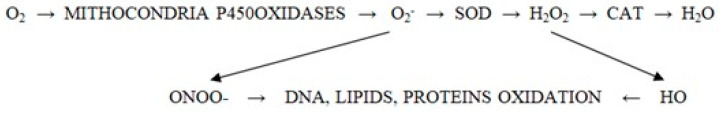
Mitochondrial O_2_ metabolism.

As illustrated by nitric oxide [NO], the superoxide anion radical [O_2_^•−^], hydrogen peroxide [H_2_O_2_], and the hydroxyl radical [OH], reactive oxygen (RO) is constituted of highly reactive molecules that are generated during physiological cellular processes. Of these, the most dangerous is the reaction with hydroxyl radicals: OH^•−^ and O_2_^•−^. The last one can be converted into relatively stable, nonradical H_2_O_2_ by superoxide dismutases (SODs)and then into water by catalase(CAT). As OH^•−^ cannot be eliminated without causing oxidative damage, its formation needs to be prevented by neutralizing the upstream sources of O_2_^•−^ and H_2_O_2_. The Fenton reaction or UV light exposure can lead to OH^•−^ formation. Due to its brief half-life (10^–9^ s at 37 °C), OH^•−^ rapidly attacks nearby molecules, causing immediate damage to proteins, nucleic acids, and lipids [48]. Mitochondrial hyperpolarization, a phenomenon within T cells in SLE, has been shown to increase the production of OH^•−^ by facilitating electron transfer to molecular oxygen, generating O_2_^•−^. O_2_^•−^ is not membrane-permeable and is converted into H_2_O_2_ by SOD2 within the mitochondria. H_2_O_2_ is further neutralized into H_2_O by CATat the expense of NADPH, but can also diffuse through membranes. Unfortunately, excess H_2_O_2_ is transformed into highly toxic OH^•−^ through the Fenton reaction. This, in turn, causes damage to lipids and other macromolecules in the immediate vicinity, resulting in the generation of diffusible and highly toxic lipid aldehydes. These aldehydes propagate oxidative stress from the mitochondria to other intracellular organelles and through the bloodstream [49,50,51] (Figure 3).

ROS are known to cause DNA damage leading to strand breaks and conformational changes. The O_2_^•−^, the most potent ROS, generated by photoexcitation, is known to selectively react with DNA. The biological consequences of O_2_^•−^-induced damage cause mutagenicity and toxicity of the genome in native DNA. Furthermore, it generates pathogenic anti-DNA antibodies [50].

Examples of ROS such as O_2_^•−^ and H_2_O_2_ have been observed to interact with NO, the production of which is frequently preceded by pro-inflammatory signals. This interaction results in the generation of reactive nitrogen species, which include the nitrosonium cation (NO+), nitroxyl anion (NO−), and peroxynitrite (ONOO−) [34].

The primary function of mitochondria is to produce ATP through oxidative phosphorylation. Beyond this core function, these organelles are implicated in a multitude of processes including the generation and detoxification of ROS, apoptosis, respiration, mitosis, and development. Disorders in these processes are clinically termed “mitochondrial dysfunction” and have been shown to modify oxygen consumption [50,51]. The joint presence of oxidative stress and mitochondrial dysfunction is known to cause damage to various molecules and cellular structures, thus resulting in alteration of the normal function of organs and systems. In turn, this can lead to the onset of conditions such as chronic tissue inflammation, dyslipidemia, and atherosclerosis [51]. It is noteworthy that oxidative stress has the potential to trigger a process referred to as immunomodulation, a factor that can contribute to the development of autoimmune diseases [52].

It is an established fact that infectious agents, especially viruses, have long been identified as playing a pivotal role in the pathogenesis of SLE. Disease flares of SLE bear a striking resemblance to the febrile illness associated with viral infection. Furthermore, immune responses in SLE have been shown to be cross-reactive between viral and self-antigens. The HRES-1/Rab4 gene is located within the 1q42 lupus susceptibility locus, a region of the genome that plays a crucial role in the development of SLE. The human endogenous retroviral element HRES-1/Rab4, which encodes Rab4A, has been identified as a key regulator in the immune system. It is notable that endogenous retroviruses have been observed to share elements of DNA with exogenous viruses. This suggests a possible role of these endogenous elements as mediators of autoimmunity in the human body. Polymorphic alleles of HRES-1/Rab4 have been found to be associated with autoantibody production and an increased risk for autoimmune diseases, including SLE and multiple sclerosis (MS) [36]. Finally, it has been determined that viral nucleic acids are more commonly detected in blood and tissue samples of patients with SLE than in healthy controls. For instance, one of the most frequent viruses associated with autoimmune disorders is the Epstein–Barr virus. In the absence of EBV proteins, quiescent B cells exhibit latency 0 [53]. However, during cell division, these cells transition to latency I upon the expression of EBNA-1, a pivotal component in the replication of the episome. On occasion, latently infected B cells undergo reactivation, a process that facilitates the reinfection of new B cells and epithelial cells. This phenomenon functions as a reservoir for viral transmission [54,55].

It is postulated that oxidative stress combines with viral infection to induce SLE by means of the following potential pathogenic mechanism: the inactivation of anti-retroviral defenses. DNA reverse-transcribed from endogenous retroviruses is metabolized by 3′ repair exonuclease 1 (TREX1), and inactivating mutations of TREX1 have been documented. Consequently, oxidative stress may potentially result in a functional TREX1 deficiency and persistent viral DNA, contributing to IC formation in SLE [55]. In addition to altering T-cell signaling and development, oxidative stress plays a role in SLE pathology via the oxidative modification of self-antigens, leading to autoimmunity. Oxidative autoantigens are known to spread throughout the circulatory system and trigger inflammation, organ damage, and comorbidities in SLE. Notable examples include the oxidation of β2 glycoprotein I (β2GPI), the cross-reactivity of autoantibodies with DNA, and the UV-induced autoantigenicity of Ro [1,56,57].

While no biomarkers of oxidative stress are currently part of routine clinical practice, it is anticipated that such a test will become available in the near future. Increased modification of serum albuminuria levels of F2 isoprostane, a derivative of lipid peroxidation, has been observed to be associated with disease activity [58,59]. Oxidized β2GPI has been found to be highly specific for the detection of antiphospholipid syndrome (APS)in the setting of thrombosis [60]. The mTOR pathway, which is involved in sensing mitochondrial hyperpolarization and oxidative stress, has been observed to correlate with disease activity. Furthermore, mTOR pathway activation has been shown to precede flares in patients with SLE [61].

## 4. The Role and Impact of Oxidative Stress on Organs in Systemic Lupus Erythematosus

The maintenance of an equilibrium between oxidants (free radicals and ROS) and antioxidants (e.g., vitamins C and E) is essential for health. However, systemic autoimmune diseases are associated with an enhanced oxidative status and mitochondrial dysfunction, which are characterized by an alteration of key physiological processes (i.e., the ability to repair vascular tissue and the control of apoptosis). In the long term, these impaired functions are directly associated with tissue and organ damage [27,62]. Dysregulation of the immune system results in the loss of tolerance, which in turn stimulates autoantibody production, inflammation, and a consequent increase in disease activity. In turn, the chronic establishment of an altered oxidative status may be associated with the onset of cardiovascular comorbidities, such as atherosclerosis and endothelial dysfunction [57]. Oxidative stress has been demonstrated to be associated with multiorgan involvement in SLE via the stimulation of undetermined IC deposition in vital organs such as the kidneys, cardiovascular system, and other organs (Table 1). In addition, the process of lipid peroxidation has a detrimental effect on the progression of end-organ injuries in SLE [58,60,63,64,65].

As previously described, NETs play a central role in the involvement of these cells in oxidative stress. The detrimental effects of NETs on endothelial cells, their activation of macrophages, and the release of MPO have all been well documented [58,64,65]. Moreover, the ability of NADPH oxidase to oxidize high-density lipoprotein (HDL) has been shown to ultimately decrease the levels of the antioxidant HDL [42]. Furthermore, oxidative stress has been identified as a pivotal factor in the formation of anti-β2GPI antibodies and thrombotic events in SLE patients with APS [58,66,67,68].

As previously stated, oxHDL has been shown to be associated with atherosclerosis in SLE, while oxidized light-density lipoprotein cholesterol has been identified as a biomarker of cardiovascular disease. The presence of ROS production has been detected in the kidneys of mice susceptible to lupus and in the circulation of patients with LN [69]. Furthermore, serum ONOO− levels have been observed to be correlated with disease activity in individuals with nephritis and Rab4A-driven mTOR activation promotes ANA production and glomerulonephritis [70]. In inflammatory diseases, including SLE, oxidized phospholipids, like 1-palmitoyl-2-arachidonoyl-sn-3-glycero-phosphorylcholine, and a series of products derived from them, build up in atherosclerotic lesions and are serum markers of oxidative stress [59,67,71,72].

Furthermore, HDL, which is susceptible to oxidation in patients with lupus, exhibits a lack of recurrent vascular protective capabilities, instead inducing a pro-inflammatory response. Levels of the oxidized β2GPI antigen demonstrate proportionality to the extent of APS and thrombotic risk (Table 1) [71,72,73,74,75].

In a similar manner, ROS have been demonstrated to be capable of affecting the integrity of DNA by altering the composition of individual nucleotide bases, thus instigating a sequence of events that lead to the formation of both single-strand breaks and cross-linking events. The continuous presence of oxidative stress over DNA has been shown to be a significant contributing factor to the development of age-related illnesses, particularly major malignancies of the colon, breast, rectum, and prostate [76]. The mechanisms that enable DNA to respond and repair itself are designed to identify genetic flaws and ensure the appropriate replacement of DNA during the cell cycle. The cell transfers the mutant genome to its descendants in the context of unrepaired lesions, and in the absence of such a process, it will be counteracted by either cell death or senescence [68]. LN has been identified as one of the most prevalent manifestations of SLE and is one of the main causes of mortality in such individuals. The deposition of ICs in the glomeruli which leads to the activation of the complement system is a key element in the pathogenesis of LN. Over time, accumulating evidence has firmly established oxidative stress as a significant contributor to renal impairment in SLE. Two biomarkers have been shown to reflect disease activity: lower vitamin C levels in the plasma of patients with LN, and a diminished ratio between glutathione (GSH) and GSSG in the kidneys of female mice models of SLE [77]. Research over several decades has demonstrated that H_2_O_2_ or ONOO− is capable of producing anti-DNA antibodies and glomerulonephritis in genetically susceptible mice [37]. Individuals with active LN exhibit an imbalanced redox state, a condition that instigates lipid peroxidation of the glomerular basement membrane and alters its integrity, thereby affecting their tubular function. The potential for oxidative stress to cause renal damage is further compounded by the production of tumor necrosis factor by the activation of neutrophils to form NETs, excessive Th17 cell differentiation, infiltrating macrophages, and secretion of the inflammatory cytokine IL-17 [48].

The occurrence of cutaneous manifestations in SLE is a recurrent complication for which the underlying etiology is yet to be elucidated. One postulation is that oxidative stress may underlie the occurrence of these diseases. Research has shown that UV light exposure in keratinocytes facilitates the release of autoantigenic Ro antibodies in blisters, thereby initiating a pathogenic autoimmune response and promoting the development of photosensitive dermatitis (see Table 1) [22,76].

Regarding liver damage, despite the fact that sub-clinical liver disease is commonplace in SLE, high levels of liver enzymes are rarely encountered. The potential role of oxidative stress in altering liver enzyme function is noteworthy, as liver enzymes are closely correlated with increases in serum NO metabolites to a certain extent [42]. Furthermore, the expression of glutamyl transferase, a nonspecific marker of liver injury, is susceptible to being induced by oxidative stress. The abnormal liver enzymes suggest a possible link between oxidative stress and liver injury. This finding supports the hypothesis of drug-induced oxidative stress, which subsequently leads to liver injury. The results of this study demonstrate that oxidative stress is a contributing factor to the specific damage of certain end-organs/systems in SLE [70,77].

The precise cause of lupus is not yet fully understood; however, there is evidence from familial clustering plus the identification of genetic susceptibility loci to suggest a hereditary component in the progression of the disease. DNA methylation, an established epigenetic mechanism that regulates gene expression, has been a subject of extensive exploration in the context of T-cell hypomethylation and its potential involvement in SLE pathogenesis [78].

SLE is associated with a few other autoimmune diseases, of which thyroid disease is categorized under “associated conditions” or conditions with increased prevalence. A study conducted in 2018 by Kuzler E. showed that of the 129 patients with SLE from a cohort, 23 (17.8%) of them had at least one coexisting autoimmune condition. The autoimmune diseases found in the highest frequencies were autoimmune thyroid disease (6 [4.7%]) [79,80]. SLE is among the top three autoimmune diseases developed by first-degree relatives of patients with celiac disease due to the fact that these diseases share common loci in the HLA region [81]. It is of great importance to differentiate between diseases which target similar structures as SLE, mainly joints. In rheumatoid arthritis (RA), an association has been demonstrated between the manifestation of the condition and a deficiency of PON1. In contrast to systemic lupus erythematosus (SLE), the pathophysiology of RA is not associated with mitochondrial metabolism and oxidative stress, but rather with a notable enhancement of antioxidant capacity in T cells. The overexpression of glucose 6-phosphate dehydrogenase has been identified as a critical factor in the production of NADPH in RA T cells. These cells produce tumor necrosis factor α inside the joint leading to bone lesions over time. The conventional process of autoantibody formation in patients with RA entails the generation of rheumatoid factor (RF), an antibody that targets the Fc region of immunoglobulin G (IgG). The release of Ig heavy chains from apoptotic B cells has been identified as a potential trigger for RF production. In psoriasis (PS), the proliferation of Th17 cells contributes to the onset of excessive inflammation, contributing to synovitis, which generates osteoclastic signaling molecules such as RANKL. Nuclear factor erythroid 2-related factor 2 (NRF2) can restrain RANKL expression and osteoclast activation if it is properly functional [82]. Recent research has linked osteoarthritis (OA) with the loss of NRF2 and the infiltration of inflammatory molecules, cytokines, and ILs. The severity of OA is also dependent on the degree of inflammatory synovitis. NRF2 inhibits osteoclast genesis, supports β-oxidation of fatty acids, and facilitates NADPH regeneration and purine biosynthesis through the pentose phosphate pathway during oxidative stress. Mice deficient in NRF2 exhibit increased RANKL expression. The vascular injury can present itself in a limited form, accompanied by calcinosis, Raynaud’s phenomenon, esophageal dysmotility, sclerodactyly, and telangiectasia, also known as CREST syndrome, which may or may not progress into a systemic form of progressive systemic sclerosis that can result in organ failure [81,82,83,84].

Due to the recent pandemic, theories of autoimmune diseases being triggered by infection with SARS-CoV-2 have been raised. Evidence has been provided that patients with a critically severe form of the infection are characterized by increased B-cell activation and secondary increased antibody-secreting cell lines [84]. Fortunately, cases of SLE after COVID-19 are rare. the presence of SARS-CoV-2 antibodies and the absence of antibodies for other viruses was affirmed in all reported patients with SLE diagnosed after SARS-CoV-2 infection. Nevertheless, further studies are required to facilitate unequivocal identification of the role of SARS-CoV-2 in SLE pathogenesis [9,81,85,86].

## 5. Therapeutic Strategies Targeting Oxidative Stress

Antioxidants can be enzymatic and nonenzymatic. The enzymatic system primarily involves CAT, SOD, glutathione peroxidase, glutathione reductase, and thioredoxin reductase mitochondrial O_2_ metabolism. GSH, the main intracellular antioxidant, is depleted, and the serine/threonine-protein kinase mTOR undergoes redox-dependent activation (Figure 3). In turn, the reversal of GSH depletion by the application of its amino acid precursor, N-acetylcysteine(NAC), reduces disease activity in lupus-prone mice. The nonenzymatic antioxidant system involves vitamin C, carotenoids, flavonoids, and GSH [52,81,85,87].

In typical conditions, T helper and T regulatory (Treg) cells have the capacity to maintain a regulated immune response. However, it has been demonstrated that oxidative stress has the capacity to inhibit anti-inflammatory Treg cell differentiation, whilst concurrently prolonging the lifespan of proinflammatory T helper cells. This, in turn, has the effect of weakening the immune response, thus resulting in tissue damage [47].

In the case of SLE, an increased level of glycolysis, alongside a decrease in lipid peroxidation, has been observed. In addition, adipokines have been found to be elevated and to exhibit a strong correlation with oxidative stress in SLE patients [65]. Leptin, an adipocyte-derived hormone, has been identified as a significant contributor to the complex interplay between adipokines and T-cell biology, particularly in the context of oxidative stress. The role of leptin in modulating T-cell proliferation and restricting the expansion of regulatory T cells (Treg) is of particular interest, as the neutralization of leptin has been observed to enhance T-cell-receptor and/or IL-2-induced Treg-cell proliferation [42].

In order to target oxidative stress therapeutically, it is necessary to consider a number of factors, including measures of prevention, exogenous triggers such as UV light, and endogenous sources such as mitochondrial function. In addition, it is also important to stimulate antioxidant mechanisms. Some interventions are more effective than others: for example, photo-resistant clothing and the application of sunscreen with a protection factor > 50 can be used to block UV light. Among the potential antioxidant therapies are NAC, which has been shown to restore GSH levels, as well as rapamycin, dietary nutrients such as vitamins, and conjugated linoleic acid (CLA). In murine models, CLA has been observed to enhance GSH synthesis, thereby reversing oxidative stress and lupus disease activity [66]. NRF2 has a pivotal role in regulating cellular REDOX homeostasis. NRF2 controls a complex transcriptional/epigenetic and post-translational network that promotes antioxidant metabolism. Itis thought to coordinate the production of antioxidants such as GSH, NADPH, and thioredoxin which neutralize pro-oxidant factors. In systemic inflammatory diseases such as sepsis, NRF2 deficiency plays an important role. Inflammation may damage proteins such as tyrosine, lysine, and histidine; these chemically modified proteins that accumulate may generate neoepitopes. These neoepitopes impair the self-tolerance of the immune system and, as a result, autoreactive T cells and autoantibodies against self-antigens are formed. In SLE, they are formed particularly against DNA, Sm, and Ro [84,88].

Elevated interferon α (IFNα) is the most common IFN biomarker associated with SLE, its role being intimately tied to cell metabolism. Glyceraldehyde-3-phosphate dehydrogenase inhibits the transcription of inflammatory messenger RNA molecules when bound to IFN; however, during glycolysis this regulation is lost, allowing inflammatory transcription to proceed. Therefore, cells that undergo constant glycolysis may exhibit increased cytokine production. This may partially explain the metabolic control of central nervous system (CNS) involvement in patients with SLE. CNS cells are highly dependent on glycolysis as a source of energy, making them especially vulnerable to oxidative stress [81].

As previously mentioned, effective antioxidant treatment may have a role not only in the therapeutic restoration of redox-mediated signaling defects but also in mitigating the toxicity of immunosuppressive therapies. mTOR is known to be activated by the relative decrease in GSH levels, the supply of which can be enhanced by supplementation with its precursor, NAC, and is diminished by oxidative stress [88].

In the field of antioxidant therapeutics, NAC has emerged as a promising candidate for its potential benefits in SLE patients [52]. Acting as a precursor to supplement GSH, NAC treatment has been documented to exert a multifaceted effect on autoantibody formation, renal inflammation, and overall survival outcomes. This medication, which is well-tolerated, has been demonstrated to reduce anti-DNA production and lupus activity via the suppression of mTOR. In addition, it has been shown to increaseFOXP3 expression in CD4+ T cells and reverse the expansion of CD4−CD8− T cells in a cohort of patients suffering from SLE. NAC’s ability to decrease lipid peroxidation, alleviate complications of the CNS, and enhance endothelial function in individuals with cerebrovascular involvement was proven. CLA has also been shown to promote GSH synthesis and reverse oxidative stress by upregulating glutamate-cysteine ligase in mice, resulting in improvements in the lupus condition [45]. mTOR, a sensor of oxidative stress in lupus T cells, has been identified as a promising therapeutic target for rapamycin [50]. Ongoing studies have confirmed the capacity of rapamycin to reduce disease activity by enhancing Treg cell expansion in SLE patients who are either resistant or intolerant to conventional immunosuppressants [45,89].

## 6. Lifestyle Modifications to Reduce Oxidative Stress

The intake of dietary antioxidants, including vitamins A, C, E, zeaxanthin, lycopene, and carotene, has been evaluated in epidemiological studies. These studies suggest that such intake may reduce the plasma levels of several oxidative biomarkers. However, no influence on the disease activity of SLE has been observed, which may be attributed to the inability of these nutrients to mediate intracellular signaling with regards to the immune system [87,90,91,92]. A series of investigations demonstrated that a methyl-poor micronutrient diet, when synergized with oxidative stress-related low T-cell levels, could potentiate the severity of lupus flares. Consequently, “methylation diet” supplementation may prove efficacious in attenuating the impairment caused by oxidative stress. The diet under analysis includes nutrients such as choline, betaine, methionine, folate, and vitamins B12 and B6 and minerals including magnesium, zinc, and sulfur. Melatonin supplementation has been associated with a reduction in cytokine production and a mitigation of oxidative stress, without a concomitant downregulation of disease activity in SLE patients. Baicalin, a validated antioxidant isolated from a Chinese herb, has demonstrated its capacity to promote Treg cell differentiation and confer protection against nephritis in murine models, potentially through antioxidative stress mechanisms, as indicated by related studies. Resveratrol, a potent antioxidant, has been observed to exert a protective effect, likely through the suppression of mTOR-mediated Th17 cell expansion [91]. Nevertheless, the ingestion of antioxidant vitamins has been found to have no influence on the risk of SLE in the general population or on disease outcomes [93]. Conversely, encouraging disease outcomes in the case of vitamin D3 have been observed in the context of autoimmunity. Supplemental vitamin D3 has been shown to reduce inflammation by inhibiting the proliferation of activated B cells, decreasing memory B cells, and causing a subsequent reduction in immunoglobulin synthesis. Moreover, it has been demonstrated to facilitate the development of regulatory T cells. These mechanisms may prove the correlation between vitamin D3 deficiency and SLE [92,93]. A plethora of other nutrients have been shown to influence regulatory T cells and cytokine production, including vitamin D, A, E, selenium, calcium, iron, magnesium, zinc, omega-3 fatty acids, phytoestrogens, and flavanols. Consequently, the supplementation of these nutrients has been shown to enhance both life expectancy and quality [94,95,96,97,98]. The intestinal barrier is influenced by dietary fiber. The fermentation of dietary fibers by intestinal bacteria results in the production of short-chain fatty acids, which in turn impacts the immune system. These are key in intestinal cell development and in the growth of regulatory T cells, which strengthens the idea that diet, microbiota, and immunity are linked [99,100]. It is hypothesized that endocannabinoids act as anti-inflammatory factors, regulating compensatory mechanisms in autoimmune diseases, due to the predominance of anti-inflammatory CB2 receptors in leukocytes. The in vitro administration of eicosanoids has been demonstrated to induce the differentiation of lymphocytes into Th2 cells. However, it is imperative to acknowledge that eicosanoids are also instrumental in the differentiation of Th1 and Th17 cells [101,102]. Consequently, the use of CB2 receptor antagonists and the genetic deletion of CB2 receptors has been observed to result in the attenuation of inflammation in animal models of PS, RA, and SLE [103].

Coenzyme Q10 (CoQ10) is a powerful antioxidant found in the membranes and lipoproteins of cells. It is produced by the body and obtained from the diet, which has made it a target for research as a therapy for mitochondrial and stress-related diseases. In vivo, the supplementation of ubiquinol in APS has been shown to modulate the expression of inflammatory and thrombotic risk markers [90,91,96]. The quinone groups in CoQ’s redox activity can be redox-reduced to the corresponding hydroquinone. Reduced CoQ, also known as ubiquinol, can be oxidized to regenerate ubiquinone. CoQ’s membrane-stabilizing properties are well documented, as is its role in providing antioxidant protection within the lipid portion of the cell membranes and plasma lipoproteins. Notably, CoQ is the only lipid-soluble antioxidant that can be produced endogenously by all aerobic organisms, ranging from bacteria to humans [90]. The heart and immune system have the highest rates of CoQ biosynthesis. These rates can be altered by some medications, including statins. CoQ10 is present in a normal diet, but its amount in dairy products is very low and it is poorly absorbed [102]. In the absence of an effective supplementation strategy, dietary CoQ10 is insufficient to support its physiological functions and does not appear to contribute to the regulation of CoQ10 levels in tissues and in plasma, which, under normal circumstances, are influenced by the rate of its endogenous biosynthesis occurring in all tissues [103]. Furthermore, the oral administration of CoQ10 has been shown to be inefficient in terms of absorption. While the presence of lipids in the diet has been shown to enhance the absorption of CoQ10, only a small proportion of the ingested CoQ10 is absorbed effectively [90,91,96]. Consequently, as the dosage of supplemental CoQ10 is increased, the percentage of the supplement that is effectively absorbed decreases. Doses that exceed the capacity for absorption have a minimal effect on efficacy [91]. High-dose CoQ10supplementationappears to be unnecessary due to its increased costs without significant benefits; nevertheless, CoQ10 has an excellent safety profile [103]. Furthermore, exogenous CoQ10 does not affect its endogenous biosynthesis, nor does it accumulate in plasma or tissues once supplementation has ceased. The relative bioavailability of CoQ10 is highly dependent on the delivery system, decreasing in the following order: nanoparticulated, solubilized, oil-emulsified, and powder [90,104,105]. Furthermore, the absorption of CoQ10 is significantly enhanced when administered in the form of ubiquinol [90,105]. Another approach to address the issue of the low bioavailability of CoQ10 involves the development of short-chain analogs, which can be effectively delivered to the desired sites to enhance CoQ10 function. Molecular analogues of interest include idebenone, a compound which has been demonstrated to enhance electron transfer chain function by circumventing deficient Complex I activity (an essential component of the electron transport chain within the mitochondria, whose activity declines in the process of aging and in the context of neurodegenerative disease), whilst concomitantly increasing the synthesis of ATP. This, in turn, has been shown to improve mitochondrial physiology. Furthermore, idebenone has been observed to result in a decline in effector memory CD4+ T cells in MRL/lpr mice. It is unclear how idebenone alters T cells, but this may involve direct effects on cell metabolism or indirect, cytokine-reducing, effects. Although idebenone reduced activated T cells, it did not affect autoantibodies and it improved endothelial-dependent vasorelaxation. The findings indicate a possible beneficial impact of idebenone on vascular health in human SLE, given the increased occurrence of endothelial dysfunction, vasculopathy, and premature atherosclerosis in this disease [88,106]. These analogs have already been assessed in clinical assays, yielding encouraging results for the treatment of numerous diseases associated with impaired mitochondrial function. Notably, those with mitochondrial tropism have been evaluated in the treatment of autoimmune diseases [107].

A diet high in fiber and reduced in sodium, protein (less than 0.6 g/kg/day), oils, and polyunsaturated fatty acids, and balanced in magnesium and folic acid is the ideal strategy according to recent research. Additionally, curcumin (120 mg–3 g/day) has been shown to have anti-inflammatory benefits in patients with PS and RA [88,104].

## 7. Downsides of the Most Frequent Therapies

The management of SLE should be focused on the treatment of acute flares, the prevention of drug-associated harms, the improvement of health-related quality of life, and the prolongation of survival. The therapeutic approach to SLE involves the combination of antimalarials (primarily hydroxychloroquine), glucocorticoids(GCs), and immunosuppressive drugs. However, these treatment plans have not been found to be universally efficacious, and exposure to them can cause significant damage [107]. Hydroxychloroquine (HCQ) is the preferred treatment for most cutaneous manifestations and has been found to be very efficacious, making it the initial treatment of choice for lupus arthritis. It is recommended for all patients with SLE given that its benefits extend beyond the management of active manifestations, including its anti-thrombotic properties and its ability to prevent flares [108]. It is imperative that patients prescribed with HCQ undergo regular ophthalmological examinations in order to monitor for the rare but irreversible maculopathy associated with this pharmaceutical agent. Following a decade of utilization, 0.1% of patients receiving HCQ have been observed to develop retinopathy [109]. In rare instances, HCQ has been observed to exert toxic effects on the heart, resulting in a QT interval prolongation [109].

GCs are widely regarded as a highly effective pharmaceutical intervention for the induction of rapid remission in patients diagnosed with SLE. However, concerns have been raised regarding the long-term use of GCs, as it has been demonstrated that they can be a primary contributing factor to the development of toxicity in these patients. The recommended dose of prednisone (1 mg/kg/day) is frequently linked to underdiagnosed and undertreated osteoporosis, which can increase the risk of osteoporotic fractures, avascular necrosis, glaucoma, hirsutism, cataracts, weight gain, acne, hypertension, and the poor management of diabetes mellitus [110]. Moreover, high-dose corticosteroids are also associated with opportunistic infections and acute psychosis. Corticosteroids are frequently used in SLE patients, many of whom are unable to reduce their dosage. It is imperative to consider and monitor the long-term adverse effects of corticosteroids. In patients receiving high-dose corticosteroids, the administration of antibiotic prophylaxis is imperative to prevent infections, as these patients are immunocompromised and thus face a significantly elevated risk of infection, which is a major cause of morbidity and mortality in SLE. The utilization of corticosteroids in doses exceeding 30–40 mg/day has been associated with a heightened risk of adverse outcomes, including infection, osteonecrosis, and even death. Conversely, pulse administration of methylprednisolone at doses < 500 mg/day for three days appears to be almost devoid of significant adverse effects. Observational studies further support these findings, demonstrating that doses of 5 mg/day are reasonably safe for long-term maintenance therapy [111,112].

## 8. Conclusions

Oxidative stress can trigger inflammation, atherosclerosis, retinopathy, nephropathy, neuropathy, cardiopathy, diabetic angiopathy, and APS. Moreover, it leads to the activation of mTOR, which has a major role in T-cell differentiation.

The potential of oxidative stress biomarkers to inform clinical decisions regarding the efficacy of antioxidant therapies is a promising avenue for future research. Significant advancements have been made in identifying biomarkers for oxidative stress. However, due to the intricate nature of the disease, it is highly improbable that a solitary biomarker can adequately reflect the comprehensive body of oxidative damage and its role in the pathophysiology of the disease. It is therefore essential to determine which particular marker, alone or in combination with others, can serve as a true indicator of the oxidative stress level in order to cause this disease. Clinical studies on SLE patients showed that using NAC reduces the oxidation of lipids, proteins, and DNA. Moreover, it reduces organ damage through mTOR blockade, and the reversal of GSH depletion reduces disease activity. However, we need to determine the mechanism by which this action is produced. Unfortunately, there is still insufficient research on the spectrum of NAC side effects when combined with conventional therapy in SLE patients. The accurate determination of biomarkers is thought to improve both the diagnosis and the management of the disease, while antioxidant therapy may open a new door for the treatment.

A more thorough characterization of the molecular and cellular origins of oxidative stress in SLE is imperative for the formulation of strategies aimed at mitigating the adverse effects of exposure. Comprehending the mechanisms of oxidative stress and the repression of deleterious pathways holds the promise of ameliorating the consequences of chronic therapy. Such an understanding has the potential to reduce disease activity and enhance the quality of life for individuals suffering from SLE.

## Figures and Tables

**Table 1:** Association between antibody titers and clinical and paraclinical manifestations (adapted from [13,14,15]).

Antibodies	Associated Manifestations
High anti-dsDNA antibodies	Malar rash and proteinuria
High anti-Ro/SSA antibodies	Photosensitivity and pericarditis
High anti-Ro antibodies + anti-dsDNA	Proteinuria (a sevenfold increased risk is observed in patients with both antibodies being positive)
High anti-La/SSB antibodies	Pericarditis (a threefold increase in the risk)
High anti-Smith antibodies	Lupus nephritis and hemolytic anemia and thrombocytopenia.
Low complement	Proteinuria

**Table 2:** The 1997 ACR (American College of Rheumatology) criteria requires 4 out of 11 criteria for the diagnosis of SLE (adapted from [19,20,21,22,23,24,25,26,27,28,29,30,31,32]).

Clinical Criteria			
Mucocutaneous manifestations (Over80% of patients with SLE suffer from mucocutaneous involvement)	Skin	1. Malar rash—the hallmark acute SLE lesion is the butterfly rash, an erythematous raised pruritic rash on the cheeks and nasal bridge which spares the nasolabial folds.	
		2. Discoid rash—chronic cutaneous lesion which may mimic squamous cell carcinoma histologically.	
		3. Photosensitivity.	
		Alopecia and Raynaud phenomenon.	
	Mucosa	4. Ulcers of the mouth and nose.	
Serosa manifestations	5. Serositis (pleuritis; pericarditis—it can cause the inflammation of any heart tissue; Libman–Sacks endocarditis, which is a sterile verrucous endocarditis with vegetations of fibrin and immune cells on the mitral valve; and myocarditis, for which an association between this condition and high anti-Ro (SSA) antibodies has been proven). Moreover, patients suffering from systemic lupus erythematosus (SLE) are predisposed to an elevated risk of developing coronary artery disease, a condition that may be attributable to coronary vasculitis or, more commonly, to widespread atherosclerosis.		
Musculoskeletal manifestations (80 to 90% of individuals with SLE suffer from musculoskeletal involvement at some point during their disease course)	6. Arthritis is a nonerosive, symmetrical inflammatory polyarthritis affecting predominantly the small joints of the hands, known as Jaccoud arthropathy. This condition results in joint capsule and ligament laxity, leading to subluxation of the metacarpophalangeal joints. These symptoms have been observed in a number of cases, and there is a possibility of these being mistaken for symptoms of rheumatoid arthritis.		
Renal disorder	7. Glomerulonephritis which is characterized by proteinuria greater than 500 mg daily or RBC in urine or casts.		
Neurologic disorders	8. Seizures and psychosis.		
Hematologic disorders	9. Anemia—over 50% of patients with SLE have this condition and can be accompanied by leukopenia of less than 4000/mm^3^ or thrombocytopenia of less than 100,000/mm^3^.		
Immunologic criteria	10. Antinuclear antibodies are very sensitive, but not very specific—ANAs are the signature of the disease and shall be the primary test performed. An immunofluorescence assay is regarded as the gold standard test for ANA. A positive ANA is observed in more than 97% of cases of SLE. It is important to acknowledge that the specificity of this antibody is only 20%, which indicates that a positive ANA does not serve as definitive confirmation of a diagnosis of SLE due to the fact that a significant proportion of the healthy population may have high levels of ANA. However, a negative ANA significantly reduces the likelihood of a diagnosis being made.		
	11.a. Anti-Smith—binds to ribonucleoproteins.		
	11.b. Anti dsDNA—binds to double stranded DNA and is seen during active disease.		
	11.c. Anti-phospholipid—usually targets the proteins which are bound to phospholipids. The presence of these antibodies leads to a hypercoagulable state → deep vein thrombosis, hepatic vein thrombosis, and stroke. As a result, lifelong anticoagulation therapy is needed. There are three types of anti-phospholipid antibodies.		Anticardiolipin—which can prove to be a false-positive for syphilis.
			Lupus anticoagulant.
			Anti-B2 glycoprotein.

**Table 3:** SLICC-2012 and ACR-2017 diagnostic ability (adapted from [20]).

Diagnostic Time	ACR1997	SLICC 2012
Clinical time	204	259
Earlier than clinical diagnosis	8	17
Later than clinical diagnosis	33	17

## Data Availability

No new data were generated.
